# Homicidal Insanity

**Published:** 1848-04-01

**Authors:** 


### HOMICIDAL INSANITY.

All medical jurists of the present day recognise a homicidal monomania which can exist *without frenzy;* that is to say, that peculiar condition of a man in which, without any derangement of the *intellect,* he is hurried away by an *irresistible inclination,* (*penchant,*) driven, impelled by a blind instinct that cannot be accounted for, to such or such an action which his reason reprobates and condemns. Beset by ideas of robbery, of incendiarism, of murder, of suicide, which he in vain strives to combat with, he feels all the horror of such desires, and yet his will is overborne; without a motive, without an interest, he robs, burns, slays, or sheds his own blood. (Pinel, Esquirol, Marc, Gall, &c.)

We feel ourselves unable to adopt, absolutely, this opinion, although it is now universally admitted by medical jurists; at least, we can receive it as correct only during the period the monomania continues before the deed is consummated; for, according to our ideas, every monomania which impels a party to the commission of such an act, is a case of monomania *with frenzy,* at least at the moment of the execution of the act. That is to say, there always, then, exists a sudden alteration or disorder, more or less remarkable, of the reason, and sufficiently powerful to deprive the individual of moral liberty, and to pervert the will to the extent of rendering it purely physical and animal. We admit, then, this impulse, this most vehement inclination, but not that it is absolutely *irresistible;* for in our opinion it becomes irresistible only at the precise moment at which the derangement of the intellect supervenes. We think the opinion that there is a sudden frenzy, an instantaneous eclipse of reason at the moment of the act, is preferable to, and more in consonance with moral science, than the hypothesis of medical jurists, who hold that the monomania, whether it be homicidal, or suicidal, or incendiary, &c., may terminate in the execution of the deed, *without frenzy* or disorder of the intellect. We repeat, we are unable to admit the theory or the principle of monomania with irresistible impulse, and without frenzy in the act, because it appears to us dangerous, inasmuch as it suspends the course of free will, destroys the morality of human actions, and tends to favour impunity of crime; for if the impulse is irresistible, and without frenzy in the act, what becomes of free will? Besides, in our opinion, a disorder of the reason will always be more easily appreciated and verified by the common sense of mankind than a perversion of the will joined to an effective lesion without frenzy in the act, which no one, after all, is able peremptorily to prove. We think, moreover, that the sudden and momentary disorder of the reason is the consequence or effect of an insufficient struggle against the wretched impulse, or of negligence in avoiding the occasions suited to its encouragement and further development. It is the circumstances, or generating causes, of the sudden or partial frenzy, which ought to determine the degree of culpability of the action of the monomaniac; because moral will is subordinate to moral liberty, as this again is subordinate to the integrity of reason.

Let us recapitulate, and say that, on the supposition the monomaniac has no reasonable interest, nor any motive sufficiently strong to urge him to an act reprobated by sound reason, such act, if completed, ought to be attributed to frenzy, and not exclusively to irresistible impulse, or to depraved will; and that we also think the doctrine of monomania without frenzy tends evidently to the overthrow of free will, or the moral liberty of human actions.

It may perhaps be objected that "if perception, judgment, and moral sensibility may be separately perverted or abolished, why should not the will, that complement of our moral and intellectual being, also be liable by itself alone, to the same perturbations, the same prostration?" This is the most plausible argument that can be opposed to our opinion; and undoubtedly we admit, with all medical jurists, and even with the generality of medical men, that the will may be more or less weakened, perverted, or depraved; but we maintain that this perversion of will *without motive* never terminates in action, unless there be at the moment of its performance, disorder or suspension of the intellect, or loss of the free will. We say without motive or interest, for every man who acts without motive or interest does not act like a reasonable person; he is in opposition to the laws of common sense—that is to say, he is a madman. The following story has been given as an instance of monomania without frenzy:—

"Mania without frenzy," Pinel says, "gave rise to a singular scene at the time of the Revolution, the recollection of which one would be glad to efface from our history. The revolutionists, during the massacres in the prisons, broke into Bicêtre Asylum, under pretext of rescuing certain victims of the old tyranny whom they represented were confined on the plea of insanity. They went armed from cell to cell; they questioned the persons in confinement, and passed on if the insanity were obvious. But one of them, who was kept in chains, arrested their attention by his sensible and rational conversation, and by his bitter complaints. Was it not detestable that he should be kept in irons and associated with madmen? He defied any one to reproach him with the least extravagant act; it was a case, he said, of the most revolting injustice, and he implored his visitors to put an end to such oppression, and become his liberators. In short, he excited among this armed gang violent murmurs and loud imprecations against the superintendent of the hospital. They compelled him to appear, and to give an account of his conduct, while every sabre was pointed against his breast; one accused him of lending himself to the most crying vexations, and another ordered him to be silent when he attempted to justify himself. He in vain stated his experience of similar cases of alienation without frenzy, though remarkable for being combined with a blind fury; their only answer was invective, and but for the courage of his wife, who shielded him with her body, he would have been assassinated. They ordered him to unchain the insane prisoner, and they carried him off in triumph, amidst redoubled cries of 'Vive la republique!' The sight of so many armed men, their loud and confused talk, their faces inflamed with wine, rekindled the madman's fury; with a vigorous arm he seized the weapon of some one near, cut away right and left; blood flew, and had not his sword been rescued from his grasp, he would, for once at least, have avenged outraged humanity.'—(Thesis of Dr. Morin.)

This is plainly a case of sudden explosion of maniacal frenzy, and instantaneous suspension of reason; and had the man committed homicide, the criminal character of a murder could not have been attached to it. There are, however, other instances more applicable to the maintenance of the opinion opposed to ours.

"A servant throws herself at the feet of her mistress, and asks permission to leave the house; she confesses that every time she undresses the child entrusted to her care —a child for whom she has all the tenderness of a mother—she experiences a desire almost irresistible to rip it open."—*Marc.*

"A kind and amiable man, of distinguished merit, daily prostrates himself at the foot of the altar, imploring the Divine Mercy to deliver him also from an atrocious inclination, for which he has never been able to give any account."—*Marc.*

"A country woman, who had not long been put to bed, and dearly loving her newborn infant, feels herself suddenly seized with a desire to cut its throat. She holds it in her arms, her eyes are fastened on it, and she is about to yield; she shakes with horror, and rushes out with the dread of being unable to control herself. She returns to suckle it,—is again agitated and distracted,—but flies. During an entire day she struggles against the ideas of destruction which unceasingly present themselves to her imagination."—*Michu, Mem. de la Monoman.-homicid.*

The following case of homicidal mania is as remarkable for its etiology as it is rare at an age so early as that of its subject. It is furnished us by M. Cerise, who has himself drawn it up for Esquirol's work on Insanity, vol. ii., p. 115.

"A young girl, about eight years of age, had manifested a determination to kill her step-mother. She was brought to M. Esquirol, and subjected by this celebrated physician to a series of questions, which she answered with sincerity, without hesitation, and with the perfect calm of innocence. She declared that she entertained no hatred whatsoever against her father's wife, that she was affected by her kindnesses, but that at the sight of her she experienced the desire of killing her. The mere presence of this woman was sufficient to excite this horrible thought. M. Esquirol, after a conversation pursued with dexterity, and a solicitude which we cannot sufficiently imitate, traces back this frightful monomania to its obscure and already forgotten origin. He discovers that some words of hatred and anger, probably accompanied with violent gestures, had been uttered some years before by her father's parents against the person whom he was about to make his second wife. The child was at that time only two years old, and this violent scene had taken place in her presence. An impression was produced,—a fact of initiative innervation corresponding to the impression, and which is renewed every time she sees her step-mother. An abnormal and vicious association is established between the efficient impression received at two years of age, and the sensorial impression produced every day by the presence of her step mother. Thence the reproduction of the initiative innervation for which this child was brought to M. Esquirol. Ignorant of any hatred, irreproachable in conscience, pure in her sentiments and will, she was moved by a blind mechanism, she obeyed an impulse at once obscure and potent, of which the murder would have been the result. What a subject for grave thought regarding the pathogeny of this form of nervous over excitement! What a subject for grave reflections on the impressibility of man in the tender years of his infancy! M. Esquirol concludes this remarkable case by calling the attention of parents to the deplorable consequences which may attend their conversation and actions in presence of their children, whose mind and heart may be thus corrupted from earliest infancy."

The reader may perceive, from this short statement, that our opinion differs from that of those medical men who admit two kinds of homicidal and suicidal monomania—one with, and the other without frenzy. We reject that species which is said to be without frenzy in the act. On the other hand, it is necessary to add, that magistrates and authorities on criminal law reject monomania absolutely, without any distinction. For instance: the advocate-general, in the case of Henriette Cornier, and M. Dupin, in the trial of Darzac; where they say that "monomania is a chimera—a mere phantom, summoned as much for the purpose of snatching the guilty from the just severity of the law, as of depriving a citizen arbitrarily of his liberty." This negative, decided, and exclusive opinion is not a whit nearer the truth; it is contradicted by hundreds of facts, which prove incontestably a homicidal or suicidal monomania, or, if you will, an impulse more or less strong and overbearing to commit criminal actions, and co-existing always with free will, or moral liberty, which is lost only at the moment of the deed. We think it would be much better to say, with M. Collard de Martigny, that "monomania is nothing but a passion which might be stifled in its birth."

Let us adopt, then, an opinion intermediate between that of physicians and that of magistrates, and say that homicidal and suicidal monomania really exists, but never without frenzy, at least, when it realizes its project or borders on the act. It is then only that the inclination, incapable of being longer subdued or repressed, can legitimately be considered as *irresistible.* M. Esquirol himself, that great authority in such matters, used, in 1821, these appropriate expressions:—

"It cannot be denied, that there are some individuals whom a fatal inclination draws on to the commission of suicide by a sort of irresistible attraction. I have never seen such persons; but I venture to think that, had people better studied the individuals who are said to have obeyed this insurmountable attraction, the motives of their determination might have been made out."—*Dict. des Scienc. Med., art. Suicide.*

But that took place in 1839. At that period, the "Gazette des Tribunaux" and other journals disclosed to France a very extraordinary fact of great importance. It is this:—In 1826, a young girl was condemned for life to the public works, for having in cold blood cut off the head of a child belonging to one of her neighbours. In the absence, says the "Gazette des Tribunaux," of all known interest in the commission of the crime, the medical men declared that the mental condition of the accused presented unequivocal symptoms of mental alienation.

Since this condemnation, several works on medical jurisprudence have placed this crime among the number of cases of homicidal monomania, and in more than one criminal affair this bloody recollection has been cited as a case in point for the defence of accused parties; but the real state of the facts came afterwards to stagger, or else to overthrow, these new theories of monomania. It would appear, according to the journals, that this woman had confessed, that after being abandoned by her lover for another person, whom he married, she had conceived the thought and project of a horrible revenge, and that she had completed her crime by butchering the child of her lover and her rival. She admitted being a little touched by the cries of the poor infant, but she kept to her purpose of vengeance. This fact, and the conclusions which have been drawn from it, joined to the avowal of the criminal, are of a nature to produce grave and serious reflections among both criminal law officers and medical jurists.

